# TGF-β1 Promotes Autophagy and Inhibits Apoptosis in Breast Cancer by Targeting TP63

**DOI:** 10.3389/fonc.2022.865067

**Published:** 2022-04-11

**Authors:** Yichao Wang, Hongsheng Lu, Zhongrong Wang, Yueguo Li, Xiaoying Chen

**Affiliations:** ^1^ Department of Clinical Laboratory Medicine, Taizhou Central Hospital (Taizhou Univesity Hospital), Taizhou, China; ^2^ Key Laboratory of Brain-Like Neuromorphic Devices and Systems of Hebei Province College of Electron and Information Engineering, Hebei University, Baoding, China; ^3^ Department of Clinical Laboratory, Tianjin’s Clinical Research Center for Cancer, Key Laboratory of Cancer Prevention and Therapy, National Clinical Research Center for Cancer, Tianjin Medical University Cancer Institute and Hospital, Tianjin, China

**Keywords:** autophagy-related genes, TGF-β1, TP63, autophagy, apoptosis, breast cancer

## Abstract

**Background:**

Breast cancer (BC) is a prevalent female cancer, which has high morbidity and mortality. However, the pathogenesis of BC has not been fully elucidated. Studies have shown that TGF-β1 plays an important role in regulating the balance between autophagy and apoptosis of tumor. We aim to clarify the specific mechanism of autophagy and apoptosis in breast cancer maintaining the tumor microenvironment.

**Methods:**

The clinical characteristics of 850 BC patients were retrieved from the TCGA database. Differentially expressed autophagy-related genes (DEARGs) between tumor and normal tissues were obtained by the Wilcox test. Through Cox proportional hazard regression analysis, the prognostic risk model was constructed and verified by the ROC curve. We used MDC staining, colony formation assay, CCK-8, flow cytometric analysis to confirm the importance of TGF-β1 on the autophagy and apoptosis of breast cancer cells. Furthermore, western blot was performed to determine the relative expression of protein. The Kaplan-Meier Plotter database was utilized to identify the prognostic value of TP63.

**Results:**

We successfully constructed a prognostic risk model of breast cancer and screened out an autophagy-related prognostic gene -TP63. We predicted that TGF-β1 and TP63 have a binding site in the JASPAR database as expected. Additionally, TGF-β1 promoted autophagy and inhibited apoptosis of breast cancer cells by inhibiting the expression of TP63.

**Conclusion:**

Our study demonstrated that the molecular mechanism of TGF-β/TP63 signaling in regulating autophagy and apoptosis of breast cancer and provided a potential prognostic marker in breast cancer.

## Introduction

Breast cancer (BC) is a common female cancer worldwide. In 2019, there were approximately 268,600 new cases of invasive breast cancer among American women, of which 41,760 women will die from the disease (1). For women, breast cancer alone accounting for 30% of female cancers (2). Breast cancer is also the most ordinary cancer among Chinese women during the last two decades. For instance, breast cancer cases in China accounted for 12.2% of newly diagnosed breast cancers worldwide and 9.6% of global breast cancer deaths, respectively (3). The common causes of breast cancer death are invasion, metastasis, and recurrence (4, 5). However, the mechanism of breast cancer occurrence and development is still unclear after years of research and extensive progress.

Autophagy can achieve cell renewal by degrading macromolecules in cells, which is conducive to maintaining the stability of the cell’s internal environment and improving cell viability (6, 7). In most cases, autophagy is considered to inhibit the occurrence of early tumors and promote the development of formed tumors (8). Apoptosis is a physiological form of programmed cell death that removes damaged cells orderly and effectively, which affects the occurrence and development of tumors. Studies have supposed that autophagy and apoptosis are related to the occurrence and development of breast cancer (9–11) and that autophagy and apoptosis play an important regulatory role in the tumor microenvironment (12–14). One of the hallmarks of cancer is that the disorder of apoptosis mechanism (15). The cytoprotective function of autophagy is achieved through negative regulation of apoptosis in many cases, and the apoptotic signal plays a role in inhibiting autophagy in turn (16). Thereby, it is worth exploring the molecular mechanism about the occurrence and development of breast cancer through regulating autophagy and apoptosis.

Transforming growth factor-β (transforming growth factor-β, TGF-β) as a key factor modulates the transformation of endothelial cells (17), which plays a dual role in promoting and inhibiting the occurrence of tumors. In the early stages of carcinogenesis, TGF-β inhibits tumorigenesis mainly by inhibiting cell growth. However, when the growth inhibitory effect of TGF-β is broken by tumor cells, TGF-β will promote the progression and metastasis of advanced tumors. TGF-β1 takes part in various cellular processes such as cell growth, differentiation, and immunity, which is necessary for regulating the tumor microenvironment (18). Studies have shown that TGF-β can induce or inhibit autophagy (19–21) and apoptosis (22) to affect the occurrence and development of tumor cells. TGF-β1, TGF-β2, and TGF-β3 are three subtypes of mammals, among which TGF-β1 shows the strongest activity. This article explores the role of TGF-β1 in the occurrence and development of breast cancer.

As a transcription factor, TP63 has a conservative basic domain structure, which belongs to the p53 transcription factor family. Compared with p53 and p73, TP63 has different functions, although they have high amino acid similarity (23). TP63 is located on chromosome 3q27-29 and consisted of 15 exons distributed over 220 kilobases. Due to two different promoters (P1 and P2), two types of proteins are produced: TAp63 and ΔNp63. TAp63 contains the N-terminal transactivation (TA) domain, and ΔNp63 is the N-terminal truncated isoform without the TA domain (24). Studies have found that TP63 is a downstream effector of the TGF-β pathway and plays an important role in primary breast cancer (25).

Based on bioinformatics analysis, this study first constructed a prognostic risk model successfully and screened the prognostic gene TP63 as one of the autophagy-related genes (ARGs) in breast cancer. We explored the interaction and effects between TGF-β1 and TP63 in breast cancer on autophagy and apoptosis. Clarifying the importance of TGF-β1 and TP63 in breast cancer cells may provide a theoretical basis and a new idea for breast cancer treatment in the future.

## Materials and Methods

### Data Collection

Corresponding clinical data and mRNA expression profiles of breast cancer patients are obtained from the TCGA database (https://portal.gdc.cancer.gov/). A total of 232 autophagy-related genes were extracted from the human autophagy database (HADb, http://autophagy.lu/Clusters/index.html).

### Analysis of ARGs

Wilcox test was used to obtain differentially expressed autophagy-related genes (DEARGs) between tumor and normal tissues. ARGs with at least two-fold change in expression level and the P value of less than 0.05 were screened out. These ARGs were deemed to be differentially expressed autophagy-related genes. Gene function enrichment analysis of DEARGs was used Gene Ontology (GO) and Kyoto Encyclopedia of Genes and Genomes (KEGG) analysis, p and q values less than 0.05 will be considered statistically significant.

### Construction of Prognostic Model Related to ARGs

After integrating the expression value of each specific gene, multivariate Cox regression analysis was used to weight the regression coefficients to successfully construct a risk score formula for each patient. Based on the risk score formula, breast cancer patients can be divided into low-risk groups and high-risk groups, and the cutoff point is the median risk score. The Kaplan-Meier method was used to assess the survival difference between the two groups. Eventually, ROC curve verified the accuracy of the prediction model. Data was divided into two groups based on clinicopathological characteristics such as age, tumor stage, tumor size, and lymph node metastasis. Age was divided into two groups for those age greater than or equal to 65 and less than 65 years old. The group of Stage was divided into Stage I & II and Stage III &IV. Tumor size for T1-2 belongs to one group, and T3-4 are another group. The group of lymph node status were divided into two groups: N0 and N1-3.

### Cells and Reagents

MDA-MB-231 and MCF-7 cells were obtained from the cell bank of Taizhou University in June 2020, and were preserved in the laboratory of Taizhou University. MDA-MB-231 and MCF-7 cells were cultivated in DMEM and MEM medium (Gibco, CA, USA) with 10% fetal bovine serum (Gibco, CA, USA), and placed at 37°C with 5% CO_2_. All cells used in this study were tested for absence of mycoplasma contamination and the authenticity of the cells was determined by short tandem repeat analysis through PCR following the instructions of ATCC in July 2020.

### MDC Staining

MDA-MB-231 and MCF-7 cells were grown in 24-well plates and cultivated at 37°C with 5% CO_2_. TGF-β1 intervention treatment was performed for 24h before staining (Solarbio, Beijing, China). After washing 3 times, the cells were incubated in the dark at room temperature for 45 min. Under a fluorescent microscope, the cells were immediately observed and photographed.

### Colony Formation Assay

MDA-MB-231 and MCF7 cells (1000 cells/well) were seeded into 6-well plates and cultivated in DMEM and MEM medium treated with TGF-β1 for 10 days. The cells were fixed with 4% polymethanol for 20 minutes and stained with 0.1% crystal violet (Solarbio, Beijing, China) at room temperature. Count and image cell colonies instantly.

### CCK-8 Assay

MDA-MB-231 and MCF-7 cells were cultivated in 96-well plates and under the conditions of 37°C and 5% CO2. On the second day, different concentrations of TGF-β1 protein (5, 10ng/ml) were added for intervention. Finally, the cell counting kit (Solarbio, Beijing, China) was used to measure the absorbance of OD_450_ at different times (24, 48 and 72h). CCK-8 (10μL) was added to each well and we should pay attention to operate in the dark during the whole process. After incubating at 37°C for 2 h, the absorbance at 450 nm was detected with a microplate reader.

### Annexin V-FITC/PI Flow Cytometric Analysis

The apoptotic cells were counted with Annexin V-FITC apoptosis detection kit (Solarbio, Beijing, China). After TGF-β1 treatment for 24 hours, cells were collected with 0.25% trypsin without EDTA and washed twice with pre-cooled PBS. Then, the cells were resuspended in 500μL of 1x binding buffer and transferred to a sterile flow cytometer glass tube. The cell suspension was incubated with 5μL Annexin V-FITC and propidium iodide (PI) solution for 15 min in dark conditions at room temperature. The cell apoptosis rates were detected by a flow cytometer (Beckman Coulter Inc, CA, USA) within 1 h.

### Western Blot

The total protein was extracted with RIPA lysis buffer (Solarbio, Beijing, China) containing protease inhibitors and phosphatase inhibitors. Then, the total protein was quantified with the BCA protein analysis kit (Solarbio, Beijing, China). The protein was separated by sodium dodecyl sulphate polyacrylamide gel electrophoresis (SDS-PAGE) and then transferred to PVDF membrane. Block the membrane in TBST buffer with 5% milk for 90 minutes, and incubate at 4°C overnight with anti-TP63 (Santa cruz, dilution 1:200), anti-P62/SQSTM1 (Abnova, dilution 1:1000), anti-Beclin1 (ABclonal, dilution 1:1000), anti-Bax (BOSTER, dilution 1:1000), anti-Bcl-2 (ABclonal, dilution 1:1000) and anti-GAPDH (ABclonal, dilution 1:40000) primary antibodies. Next, the secondary antibody (EarthOx, dilution 1:40000) labeled with horseradish peroxidase (HRP) was used for 2 hours at room temperature. A chemiluminescence imaging system (Amersham Imager 680, GE, USA) was used to detect the band intensity. The results were normalized to GAPDH, and Image J (National Institute of Mental Health, Bethesda, USA) was applied for band density analysis.

### Statistical Analysis

Use SPSS26.0 and R3.6.3 software for statistical analysis. The log-rank test was used for comparison. Established a Cox proportional hazards model by Multivariate analysis. Kaplan-Meier Plotter was used to draw survival curves. Student’s t-test and one-way ANOVA were used to analyze the statistical differences between the two groups and multiple groups, respectively. The experiment was repeated at least three times. P<0.05 was considered statistically significant.

## Results

### Screening of DEARGs

**Figure 1:**
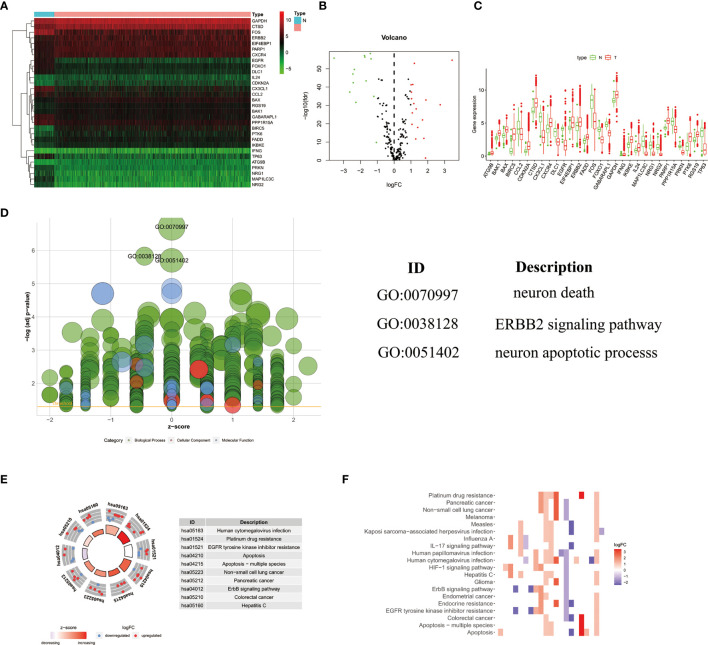
The heatmap **(A)** and volcano map **(B)** showed 30 differentially expressed autophagy-related genes in breast cancer tissues. Red represented high expression level, green represented low expression level, and black represented no significant difference. **(C)**The box plot showed the expression patterns of 30 differentially expressed ARGs. Red dots indicated tumor tissues, and green dots indicated normal tissues. **(D)** GO analysis showed the biological processes, cellular components, and molecular functions involved in ARGs; the green, pink, and blue bubbles represented biological processes, cellular components, and molecular functions, respectively. **(E, F)** The potential enrichment pathways of ARGs in breast cancer were obtained by KEGG analysis.

The detailed workflow for the construction of the prognostic risk model was presented in **Figure S1**. There are 1085 cases of clinical data were collected from the TCGA database (https://portal.gdc.cancer.gov/). Finally, a total of 850 cases of primary breast cancer with follow-up time of more than 1 month and a complete gene expression profile were included for follow-up analysis. We extracted the expression value of 232 ARGs. According to the criteria of p<0.05 and log_2_(fold change)>1, we lastly obtained 17 up-regulated DEARGs and 13 down-regulated DEARGs (**Figures 1A, B**). The box plot showed the expression patterns of 30 DEARGs between tumor and normal tissues (**Figure 1C**).

### Functional Enrichment of DEARGs

The functional enrichment analysis of 30 differentially expressed ARGs provides a biological understanding of these genes. It can be seen from GO and KEGG analysis that DEARGs are mainly involved in autophagy, apoptosis signaling pathway, ERBB2 signaling pathway, etc. (**Figures 1D–F**).

### Construction and Verification of the BC Prognostic Risk Model

**Figure 2:**
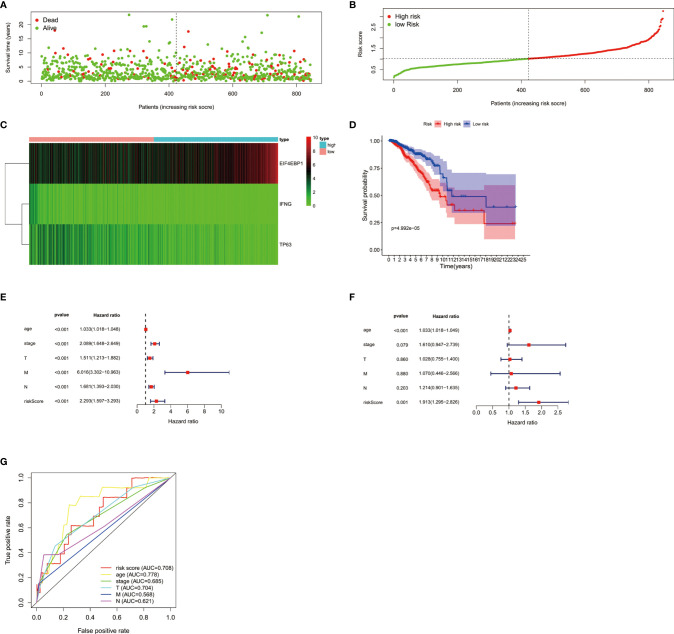
**(A)** The distribution of prognostic indicators in different groups of breast cancer patients. **(B)** The distribution of risk score in different groups of breast cancer patients. **(C)** Heatmap of the expression profile of DEARGs. **(D)** Survival curves of breast cancer patients in the high and low risk groups. The forest plots showed univariate Cox regression analysis **(E)** and multivariate Cox regression analysis **(F)** in breast cancer. **(G)** The survival-dependent receiver operating characteristic (ROC) curve confirmed the prognostic significance of risk score based on ARGs.

In order to analyze the role of ARGs in the prognosis of breast cancer, we first screened the ARGs that are significantly related to the prognosis of breast cancer. Using univariate Cox regression analysis, a total of 4 autophagy-related genes (EIF4EBP1, IFNG, NRG1, TP63) were significantly correlated with overall survival (OS) of breast cancer (**Table 1**). Multivariate Cox regression analysis showed that a total of 3 genes (EIF4EBP1, IFNG, TP63) were significantly related to the prognosis (**Table 2**). The results showed the distribution of ARGs signals in the TCGA data set (**Figure 2A**), the risk score of different groups of patients (**Figure 2B**), and the heatmap of the included ARGs expression profile (**Figure 2C**). The K-M survival curve revealed the different survival time between the high-risk group and the low-risk group, verifying the performance of the prognostic model in predicting OS in BC patients. We observed that the survival time of the low-risk group was significantly higher than the high-risk group (**Figure 2D**), and there was a significant difference between the two groups(P<0.05). After adjusting the clinicopathological characteristics such as age, tumor stage, tumor size, and lymph node metastasis, univariate analysis (HR=2.293, 95%CI, 1.597~3.393; P<0.001; **Figure 2E**) and multivariate analysis (HR= 1.913, 95% CI, 1.295~2.826; P=0.001; **Figure 2F**) indicated that the risk value is an independent prognostic indicator for breast cancer patients. The AUC value for 5 years of risk score is 0.708, which is significantly higher than tumor stage (0.685), metastasis status (0.568), and lymph node status (0.621), which indicated that the prognostic risk index based on ARGs had certain potential in survival prediction (**Figure 2G**).

**Table 1:** Univariate cox regression analysis identified 4 ARGs related to the BC risks.

Genes	HR	95% CI	p value
EIF4EBP1	1.224	1.051-1.426	0.009
IFNG	0.601	0.386-0.934	0.024
NRG1	0.642	0.418-0.985	0.042
TP63	0.795	0.658-0.959	0.017

**Table 2:** Multivariate cox regression analysis identified 3 ARGs that are independent factors for BC risks.

Genes	Co-efficient	HR	95% CI	p value
EIF4EBP1	0.191	1.211	1.039-1.412	0.015
IFNG	0.565	0.568	0.367-0.880	0.011
TP63	0.197	0.821	0.680-0.992	0.041

### Clinical Correlation Analysis of TP63

**Figure 3:**
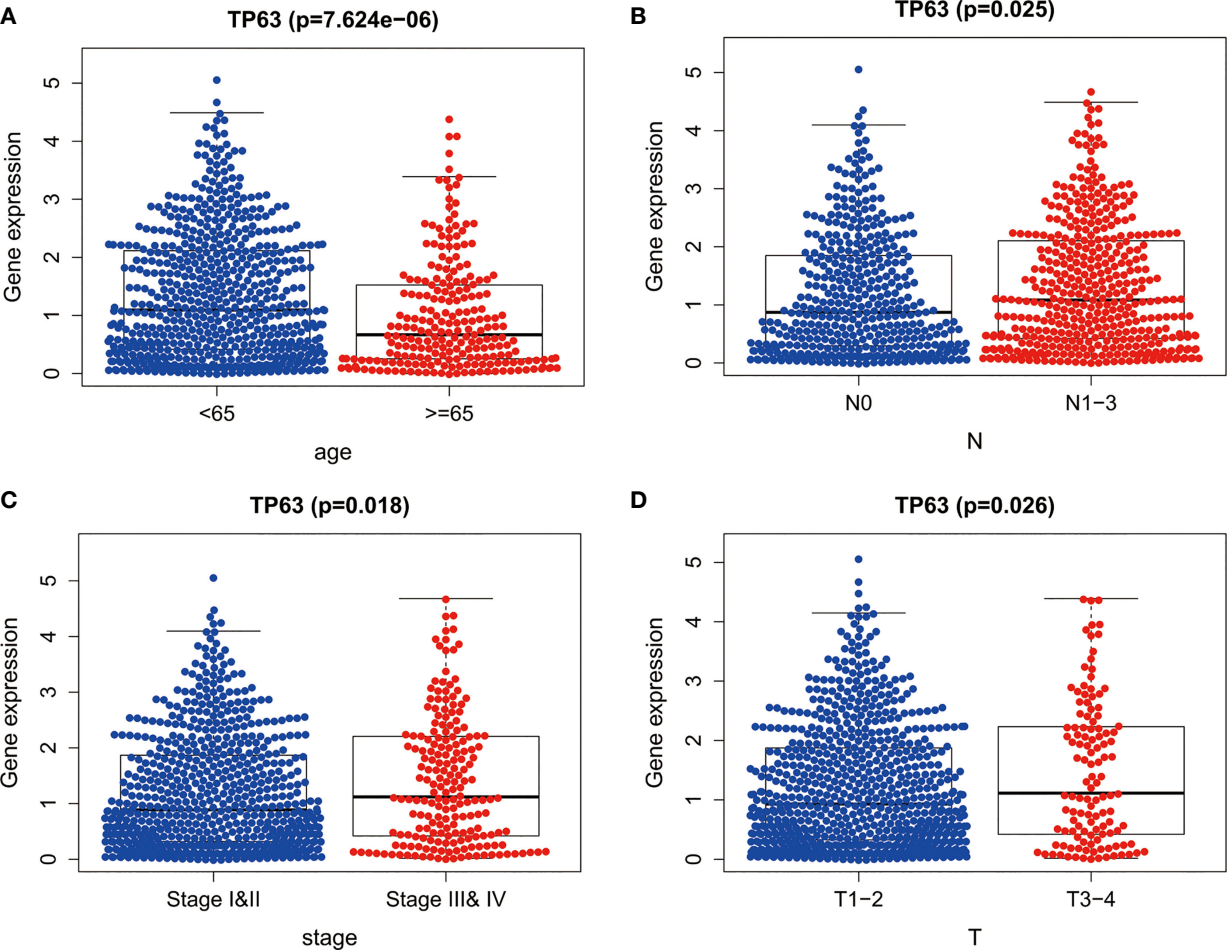
The expression of TP63 between different clinical features of breast cancer. The P values of **(A)** age, **(B)** lymph node metastasis, **(C)** TNM stage and **(D)** tumor size between the two groups were all less than 0.05.

The clinical significance of TP63 in breast cancer was evaluated by analyzing the correlation between the expression of TP63 and clinical parameters. These parameters demonstrated that TP63 was significantly related to patient’s age (**Figure 3A**), lymph node metastasis (**Figure 3B**), TNM stage (**Figure 3C**), and tumor size (**Figure 3D**).

### TGF-β1 Promoted Autophagy in Breast Cancer by Targeting TP63

**Figure 4:**
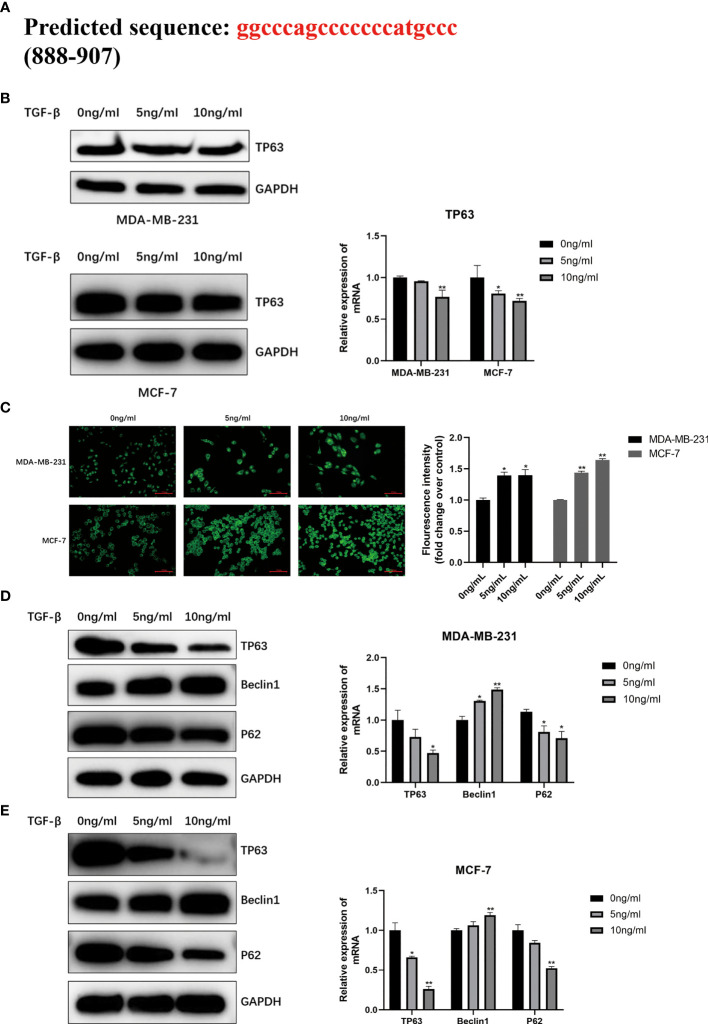
**(A)** Predicted the TGF-β1 target TP63 potentially by using JASPAR database (http://jaspar.genereg.net/) and verified the TGF-β1 is negatively correlated with TP63 **(B)**. MDC staining was used to analyze autophagy **(C)**. When MDA-MB-231 cells and MCF-7 cells were induced by TGF-β1 for 24 hours, MDC staining was performed in the dark and observed under a fluorescence microscope immediately. Fluorescence intensity was quantified by ImageJ and shown as mean ± SD. The expression level of the autophagy-related proteins and TP63 in MDA-MB-231 cells **(D)** and MCF-7 cells **(E)** with TGF-β1 induced. GAPDH was used as an internal control. Quantitative analysis of TP63, Beclin1, and P62 are expressed as the mean ± SD (*p < 0.05, **p < 0.01).

As shown in **Figure 4A**, the JASPAR database (http://jaspar.genereg.net/) predicted that TP63 was located as a transcription factor on the TGF-β1 promoter sequence, and there was a potential binding site between them. In order to illuminate the correlation between TGF-β1 and TP63, we detected the expression level of TP63 in breast cancer cells with TGF-β1 induced. We clearly observed that TGF-β1 inhibits TP63 in breast cancer cells (**Figure 4B**).

MDC staining was performed on MDA-MB-231 and MCF-7 cells to directly observe the autophagosomes. The results confirmed that TGF-β1 is related to the degree of autophagy. We can find that the two kinds of cells treated with TGF-β1 (5ng/ml, 10ng/ml) showed stronger fluorescent spots (**Figure 4C**) by MDC staining, that is, highly autophagy is activated in treated cells. Moreover, TGF-β1 inhibited P62 and TP63 protein levels in MDA-MB-231 (**Figure 4D**) and MCF-7 cells (**Figure 4E**), and increased Beclin1 protein levels. It is obvious that TGF-β1 enhanced the autophagy level in breast cancer cells by inhibiting TP63.

### TGF-β1 Inhibited Apoptosis in Breast Cancer by Targeting TP63

**Figure 5:**
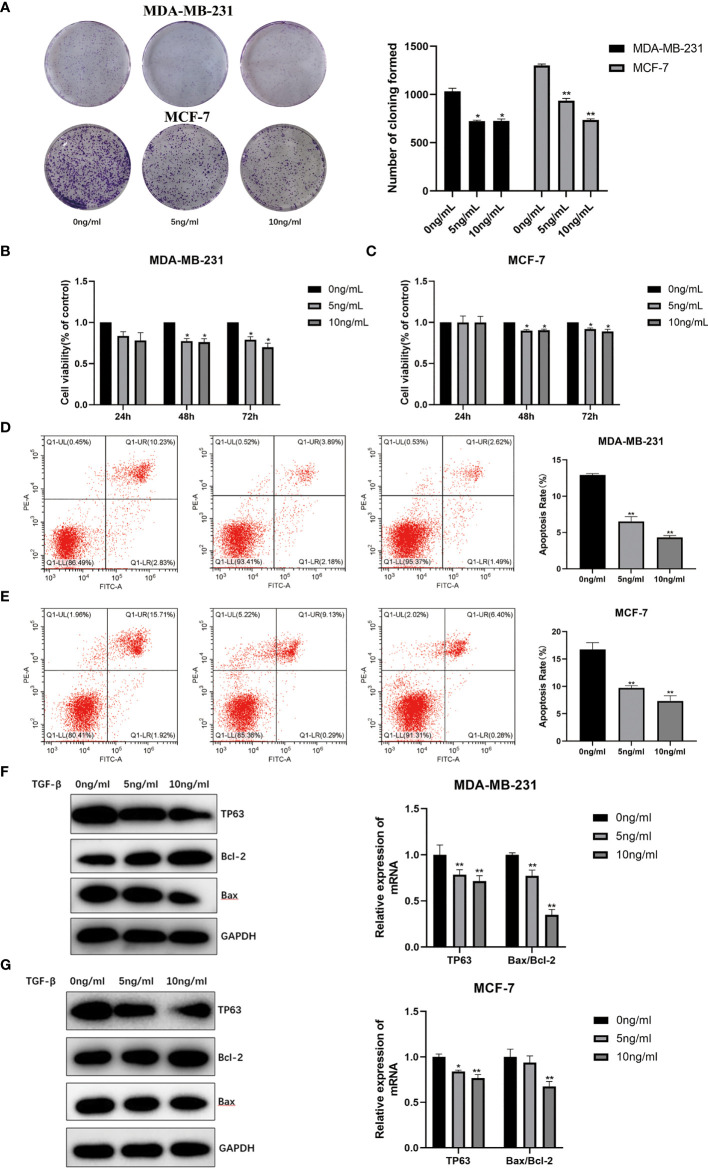
**(A) **Proliferation ability of MDA-MB-231 and MCF-7 induced by TGF-β1 by plate cloning experiment. The effect of TGF-β1 on the proliferation of MDA-MB-231 cells **(B)** and MCF-7 **(C)** cells were analyzed by CCK-8 (*p < 0.05, **p < 0.01). Analysis of the effect of TGF-β1 on the apoptosis of MDA-MB-231 cells **(D)** and MCF-7 **(E)** cells by Annexin V-FITC/PI stain flow cytometry (*p < 0.05, **p < 0.01). The expression level of the apoptosis and TP63 proteins in MDR-MB-231 cells **(F)** and MCF-7 **(G)** cells with TGF-β1 induced. GAPDH was used as an internal control. Quantitative analysis of TP63, Bcl-2 and Bax are expressed as the mean ± SD. *, **p < 0.01 vs. control group.

In order to further explore the relationship between TGF-β1 and the ability of proliferation in breast cancer cells, TGF-β1 protein (5, 10ng/ml) was acted on MDA-MB-231 and MCF-7 cells respectively. Compared with the control group, we observed that the number of cell colonies with TGF-β1 treatment was much smaller (**Figure 5A**). The cell proliferation abilities after 24, 48, 72h induced by TGF-β1 were tested by the CCK-8 method. As shown in **Figure 5**, TGF-β1 inhibited the proliferation of MDA-MB-231 (**Figure 5B**) and MCF-7 (**Figure 5C**) cells in a concentration and time-dependent manner.

The Annexin V-FITC/PI flow cytometric analysis was used to detect the apoptosis rates of MDA-MB-231 and MCF-7 cells with TGF-β1 treatment. The apoptotic rates of MDA-MB-231 cells (**Figure 5D**) with TGF-β1 treated in each group were (12.93 ± 0.18) %, (6.54 ± 0.66) %, (4.31 ± 0.28) %, and the apoptosis rates of MCF-7 cells (**Figure 5E**) with TGF-β1 treated in each group were (16.8 ± 1.23) %, (9.72 ± 0.42) %, (7.35 ± 0.94) %, respectively. From the results, we can find that the apoptosis rates of MDA-MB-231 and MCF-7 cells were inhibited by TGF-β1 (p<0.05). In terms of WB results, the expression level of TP63 and Bax were decreased, while the expression level of Bcl-2 gradually was increased with the increasing of TGF-β1 (**Figures 5F, G**). The results supposed that TGF-β1 can inhibit the ability of cell apoptosis by inhibiting TP63.

### Prognostic Value of TP63 in Breast Cancer

**Figure 6:**
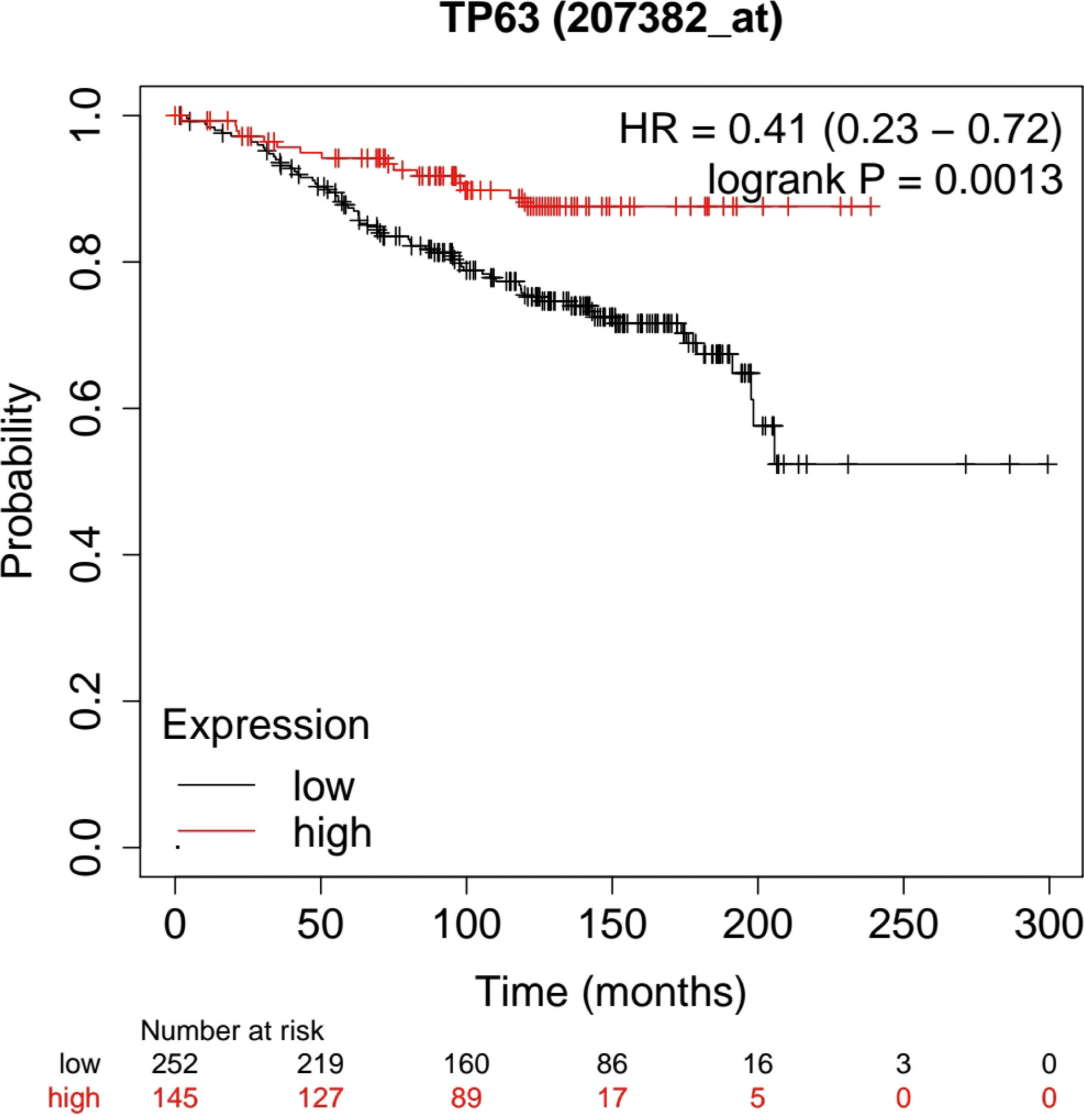
Kaplan-Meier analysis was used to determine the prognostic role of TP63 in breast cancer. The prognostic potential of TP63 expression in breast cancer (p < 0.05).

Based on the Kaplan-Meier Plotter database (http://www.kmplot.com/analysis/), the prognostic potential of TP63 in breast cancer was explored. A total of 397 untreated breast cancer (BC) patients were included in the analysis. The results showed that the expression level of TP63 in breast cancer was positively correlated with the overall survival (OS) of patients [HR=0.41 (0.23-0.72), p=0.0013] (**Figure 6**). According to the different clinicopathological characteristics in the Kaplan-Meier Plotter database, the relationship between TP63 and the prognosis in breast cancer was explored. As shown in **Table 3**, it can be seen that TP63 is related to ER, HER2, subtype, Grade stage, and TP53 status in breast cancer patients, and the difference is statistically significant.

**Table 3:** Correlation between TP63 expression and different clinical pathological factors by Kaplan-Meier plotter.

Clinicopathological characters	OS (Overall Survival)		
	N	HR	p value
Total	397	0.41(0.23-0.72)	0.0013
ER			
positive	259	0.25(0.1-0.58)	0.00049
negative	96	0.54(0.22-1.34)	0.18
unknown	42	–	–
HER2			
Positive	57	2.03(0.66-6.2)	0.21
negative	340	0.44(0.26-0.75)	0.0021
Intrinsic subtype			
basal	58	0.44(0.13-1.53)	0.18
luminal A	225	0.36(0.16-0.8)	0.0095
luminal B	95	0.26(0.07-0.88)	0.02
HER2^+^	19	–	–
Grade			
1	95	0.32(0.08-1.25)	0.085
2	176	0.33(0.17-0.66)	0.00091
3	121	1.83(0.91-3.65)	0.084
unknown	5	–	–
TP53 status			
mutated	29	0.25(0.05-1.29)	0.073
wild type	111	0.2(0.06-0.65)	0.003
unknown	257	–	–

## Discussion

Although the treatment for breast cancer has made great progress in cancer research, it is still a major health problem that plagues all mankind. In China and even all over the world, breast cancer is a common cancer among women and its morbidity and mortality are increasing gradually (26–29). Recently, it has been reported that autophagy is related to breast cancer invasion and metastasis (30), and even drug resistance (31, 32). Interestingly, there are numerous studies have found that autophagy can be considered as a new target for breast cancer treatment, but more in-depth researchs are still needed to clarify the specific mechanism of autophagy in the development of breast cancer. Breast cancer is a kind of highly heterogeneous type of cancer with high invasion and metastasis ability. The current standard systemic therapies (hormonal, cytotoxicity, and HER2 targeted therapy) are not suitable for every patient (33). Therefore, it is very necessary to find new targets for the treatment of breast cancer. The 5exploration of ARGs may provide a more adequate theoretical basis for breast cancer prognosis prediction and treatment.

From the current study, we firstly screened out prognostic-related ARGs based on the clinical information of 1085 breast cancer in the TCGA data by bioinformatics analysis. Using multivariate Cox regression analysis, we confirmed three ARGs (EIF4EBP1, IFNG, and TP63). These ARGs have shown a strong ability in the prognosis of breast cancer or other malignant tumors. EIF4EBP1 is a direct target of mTOR and a key effector of protein synthesis. Loss of EIF4EBP1 is associated with poor overall survival in patients with head and neck squamous cell carcinomas (HNSCC) (34). Also, EIF4EBP1 frequently increased in breast cancer, which is considered to be an indicator of poor prognosis and resistance to endocrine therapy (35). SK Ganapathi et al. reported that the expression level of IFNG in peripheral blood mononuclear cells (PBMCs) of patients with recurrent colorectal cancer (CRC) was significantly lower (36). It is reported that TP63 can predict the progression and survival of bladder cancer, kidney cancer, low-grade glioma, and skin cancer (37, 38). We successfully constructed a breast cancer prognostic risk model based on ARGs which can effectively evaluate the prognosis of breast cancer patients.

Based on the literature (39–41) and the clinical correlation analysis, we eventually screened out an autophagy-related gene-TP63. Adorno M et al. have reported that TGF-β1 promoted the invasion and metastasis of breast cancer by inhibiting the expression of TP63 (42). It has been reported that TGF-β may play an important role in the progression of breast cancer through ΔNp63 or inducing/inhibiting autophagy, and TGF-β-regulated miRNA network is crucial for regulating the expression of ΔNp63 in breast cancer progression (25, 43, 44). In addition, we predicted a binding site between TGF-β1 and TP63 by JASPAR database. WB results verified that TGF-β1 can inhibit the expression of TP63, and there were negatively correlated between TGF-β1 and TP63. This result was consistent with the previous research. The balance between autophagy and apoptosis is closely related to the tumor microenvironment (45–47). However, studies in regulating autophagy and apoptosis in breast cancer about TGF-β1 and TP63 have not been reported in word. Therefore, the effects of TGF-β1 and TP63 on autophagy and apoptosis in breast cancer cells were explored in this study.

Chen Liang et al. demonstrated that TGF-β1 induced autophagic flux in pancreatic ductal adenocarcinoma (PDAC) (48). TGF-β1 activated cancer-associated fibroblasts to promote breast cancer invasion, metastasis, and epithelial-mesenchymal transition by autophagy (49). These studies showed that TGF-β1 can promote autophagy in the development of cancer. In our research, the autophagy level was determined by MDC staining and WB, which showed that the autophagy level of breast cancer cells was affected by TGF-β1 and was increased when TGF-β1 induced. We found that the autophagy levels of MDA-MB-231 and MCF-7 cells treated with TGF-β1 were improved, and there was a concentration-dependent relation between TGF-β1 and autophagy levels. Furthermore, WB results proved that the expression of TP63 was decreased with TGF-β1 induced. The above results proved that TGF-β1 can target TP63 to promote autophagy in breast cancer cells.

Autophagy and apoptosis are inseparable (50, 51). Therefore, TGF−β1 can also regulate tumor progression by affecting apoptosis. A study has suggested that TGF-β1 can influence cervical cancer cell proliferation and apoptosis and TGF-β is a potential target for cervical cancer therapeutics (52). TGF−β1 treatment protects tumor cells from various apoptotic stresses, including 5−fluorouracil, etoposide, and γ−irradiation in human colon cancer (53). TGF-β1 even plays an important role in the apoptosis of breast cancer (54). From the colony formation experiment, the number of cell clones in the experimental group treated with TGF-β1 was less than that in the control group. According to CCK-8 results, TGF-β1 had no effect on the proliferation ability of breast cancer cells until 48h. There was a time-dependent and concentration-dependent relation between TGF-β1 and the proliferation ability of breast cancer cells. In addition, we used flow cytometry to detect the apoptosis rates of MDA-MB-231 and MCF-7 cells after TGF-β1 treatment. The results of Annexin V-FITC/PI flow cytometric analysis showed that TGF-β1 inhibited the apoptosis level of MDA-MB-231 and MCF-7 cells. Similarly, TGF-β1 was determined to inhibit apoptosis of breast cancer cells by targeting TP63 from the WB results. In the future, we need to further explore about how autophagy and apoptosis regulating the tumor microenvironment *via* the TGF-β1/TP63 signaling pathway in breast cancer.

A large number of studies have reported that TP63 can be used as a prognostic factor for cancers such as salivary gland adenoid cystic carcinoma, anaplastic large cell lymphoma, and squamous cell carcinoma (55–58). The following analysis demonstrated that TP63 as an autophagy-related gene is a low-risk factor for the prognosis of breast cancer, that is, the lower the expression of TP63, the worse the prognosis of breast cancer. One point in our result is that TP63 was related to advanced Clinicopathological indicators from bioinformatics analysis. Possibly due to two different promoters of TP63 (P1 and P2), and two types of proteins are produced: TAp63 and ΔNp63. TP63 has two subtypes, but which one responsible for breast cancer is not specified. And also, breast cancer has many subtypes. In the bioinformatics analysis, it is not clearly that which subtype plays the important role in the database. In future studies, we will focus on this doubt. We explored that the prognostic value of TP63 in breast cancer by the Kaplan-Meier Plotter database. Therefore, TP63, as an important prognostic gene, may be expected to become a potential prognostic biomarker.

In summary, our study constructed a prognostic model related to autophagy in breast cancer, and TP63 was screened out as a key factor in the prognostic model. TGF-β1 can promote autophagy and inhibit apoptosis on MDA-MB-231 and MCF-7 breast cancer cells by targeting TP63 to affect the occurrence and development of breast cancer. This may provide a new theoretical basis to the research on the mechanism of occurrence and development in breast cancer, as well as the value of prognostic prediction.

## Data Availability Statement

The original contributions presented in the study are included in the article/**Supplementary Material**. Further inquiries can be directed to the corresponding authors.

## Author Contributions

Data curation, YW. Formal analysis, YL. Funding acquisition, YW and XC. Investigation, ZW. Software, HL. Writing – review and editing, YW and XC. All authors contributed to the article and approved the submitted version.

## Funding

This work was supported by the Natural Science Foundation of China (grant number 81902138), The Project of Public Welfare Technology Application of Zhejiang Province (grant number LGF22H200016) and the Hangzhou Medical and Health Science and Technology Project (grant no. B20220609).

## Conflict of Interest

The authors declare that the research was conducted in the absence of any commercial or financial relationships that could be construed as a potential conflict of interest.

## Publisher’s Note

All claims expressed in this article are solely those of the authors and do not necessarily represent those of their affiliated organizations, or those of the publisher, the editors and the reviewers. Any product that may be evaluated in this article, or claim that may be made by its manufacturer, is not guaranteed or endorsed by the publisher.

## Supplementary Material

Supplementary Figure S1 The workflow for the construction of the prognostic risk model about autophagy-related genes in BC.
