# Supplementary figures and images for: TGF-β1 Promotes Autophagy and Inhibits Apoptosis in Breast Cancer by Targeting TP63
Source: Front Oncol. 2022 Apr 11;12:865067. doi: 10.3389/fonc.2022.865067 (PMC9035888; doi:10.3389/fonc.2022.865067)

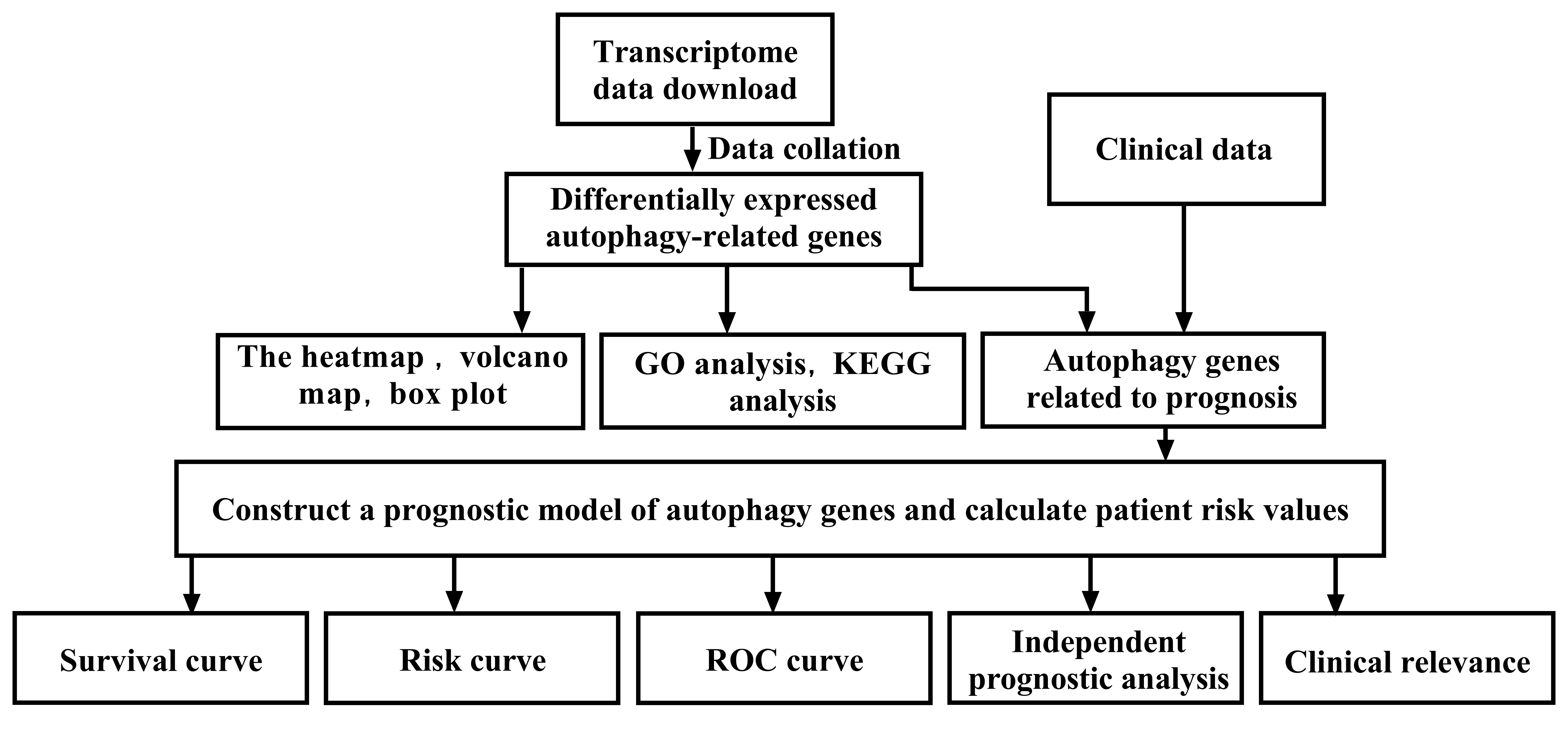

Supplement: Supplementary Figure S1 — The workflow for the construction of the prognostic risk model about autophagy-related genes in BC. [file Image_1.tif]
